# How Do Technological Artefacts Embody Moral Values?

**DOI:** 10.1007/s13347-020-00401-y

**Published:** 2020-05-15

**Authors:** Michael Klenk

**Affiliations:** Delft University of Technology, 2628BX Delft, The Netherlands

**Keywords:** Ethics of technology, Value embedding, Artefacts, Moral value, Response-dependence

## Abstract

According to some philosophers of technology, technology embodies moral values in virtue of its functional properties and the intentions of its designers. But this paper shows that such an account makes the values supposedly embedded in technology epistemically opaque and that it does not allow for values to change. Therefore, to overcome these shortcomings, the paper introduces the novel Affordance Account of Value Embedding as a superior alternative. Accordingly, artefacts bear affordances, that is, artefacts make certain actions likelier given the circumstances. Based on an interdisciplinary perspective that invokes recent moral anthropology, I conceptualize affordances as response-dependent properties. That is, they depend on intrinsic as well as extrinsic properties of the artefact. We have reason to value these properties. Therefore, artefacts embody values and are not value-neutral, which has practical implications for the design of new technologies.

Technology is value-laden. That is, development and deployment of technology can plausibly undermine or promote specific values (van den Hoven 2005). Moreover, beyond merely influencing what people value, technological artefacts might themselves embody values (Kroes 2012; Houkes and Vermaas 2010; Kroes and Verbeek 2014; Winner 1980).^1^ Accordingly, an assault rifle like the AK47 would be morally good or bad, quite independently of how the rifle is used. While the claim about technology’s mediating role is uncontroversial, the second claim about technology’s capability to bear value is subject to considerable debate. Defenders of the value-neutrality thesis deny that technology embodies value (Pitt 2014; Peterson and Spahn 2011). Exemplifying the value-neutrality thesis, David Sarnoff, the founder of the National Broadcasting Company, claimed (cited in McLuhan 1994):

We are too prone to make technological instruments the scapegoats for the sins of those who wield them. The products of modern science are not in themselves good or bad; it is the way they are used that determines their value.

Defenders of the value-neutrality thesis concede of course that actions or events that involve artefacts can have value, but they deny that the artefact itself can have value. However, in the light of efforts to innovate responsibly, and the intuition that some artefacts, such as the AK47, are inherently more problematic than others, such as a sponge, the value-neutrality thesis is frequently denied. Some claim that artefacts have something like interactive moral agency (Verbeek 2006, 2014; cf. Peterson and Spahn 2011 for critical discussion), and therefore are not value-neutral, while others argue that artefacts can embody value independently of whether they are (relevantly similar to) moral agents or not (Flanagan et al. 2008; van de Poel and Kroes 2014; Brey 2014; Houkes and Vermaas 2010).^2^ I will be exclusively concerned with the latter claim in this paper.^3^

This paper aims at contributing to the project of finding a suitable account of value embedding for technological artefacts. It does so by criticizing what is arguably the most developed account of value embedding for technological artefacts, due to Van de Poel and Kroes (2014).

Van de Poel and Kroes argue that an artefact embodies a given value provided that its designed properties contribute to that value. I call this the intentional history account of value embedding (IHAVE).^4^ I will show that this account raises insurmountable epistemic and metaphysical issues. Epistemically, IHAVE makes it difficult to discern whether an artefact is morally good or bad. The problem is that design intentions (to wit, the intentions that according to IHAVE partly determine whether the artefact is good or bad), are not directly perceivable. Since design intentions underdetermine design choices (to wit, the physical properties of the object), design intentions are also not indirectly perceivable. Therefore, if IHAVE were true, we would be unable to know what value an artefact embodies. Metaphysically, the problem is that embedded values cannot change if IHAVE were true. According to IHAVE, an artefact’s value is partly determined upon its creation. However, artefacts get appropriated for uses unforseen by the designer, and so their value changes perceptively. This cannot be accounted for by IHAVE. Though this is no conclusive proof that IHAVE fails, I conclude that it is a strong reason against IHAVE.

However, rather than endorsing the value-neutrality thesis, I turn to the concept of an affordance, which has its roots in ecological psychology and is now increasingly used in moral anthropology, to develop an alternative affordance account of value embedding. I argue that artefacts have affordances as relational properties. Based on a discussion of the roots of the concept ‘affordance’ in ecological psychology, and its use in anthropology, I argue that affordances are best conceived of as response-dependent properties. Finally, I argue that response-dependent properties can be finally valuable. Hence, since artefacts bear affordances, they can be finally valuable, too. This shows that the value-neutrality thesis is false, and it offers a first sketch of an account that makes sense of why this is the case.

Apart from its original contribution to the philosophy of technology, the paper’s conclusion should also be of relevance to scholars in digital ethics, many of whom are busy discussing the value of new technologies such as artificial intelligence. The affordance account of value embedding provides them with a way to make sense of the potentials and problems with new technology beyond their use.

## The Intentional History Account of Value Embedding

According to van de Poel and Kroes (2014), a technological artefact embodies the value it was designed to contribute to. They give the example of a sea dyke. Suppose the dyke was designed for safety (to protect people from floods), and it actually does contribute to the value of safety (by protecting people from floods). For these reasons, they write, the dyke embodies the value of safety. More formally, they define value embedding as follows (van de Poel and Kroes 2014):

Value Embedding According to IHAVE: Artefact x embodies value V if the designed properties of x have the potential to achieve or contribute to V due to the fact that x has been designed for V.

In other words, the conditional states a necessary condition for an artefact to embody a value: the artefact must potentially contribute (in some sense) to the value and it must be doing so in virtue of being so designed.^5^

The notion of value at play here as well as the notion of contributing to a value and the concept of design at play in the conditional need clarification. The kind of value that van de Poel and Kroes see embedded in artefacts is extrinsic final value (van de Poel and Kroes 2014). This is based on a distinction of value along two dimensions: the grounding of the value (intrinsic, extrinsic) and the relationality of the value (final, instrumental). An object that has extrinsic final value possesses final value (i.e. it is valuable in itself), but the fact that it is valuable in itself depends on relational properties (which make it extrinsic rather than intrinsic value).^6^ For example, a wedding ring may possess extrinsic final value: the ring is finally valuable to someone, but that is only because of its relational properties (it is your ring, for example). As van de Poel and Kroes note, the existence of extrinsic final value is controversial. However, they follow Korsgaard (1983) in supposing something can possess final value even if it is not good in itself; if, for example, circumstantial factors determine whether something is good. Korsgaard gives the example of human happiness understood in a Kantian way: happiness is good for its own sake, even though happiness is not good in itself but only if brought about by goodwill (van de Poel and Kroes 2014).

The restriction to extrinsic final value is crucial for the intentional history account. It allows its proponents to say that artefacts bear value only if the properties that allow the artefact to contribute to the value are *designed* properties. The intrinsic features of the artefact (e.g. its physical structure) thus do not exhaustively determine the artefact’s value, but its extrinsic features, the intentions of the artefact’s designers, necessarily contribute to its value, too.

The previous point relates to the question of why the notion of design intentions is so vital for IHAVE. According to IHAVE, the artefact’s contribution to a given value does not suffice for the value to be embodied by the artefact. Instead, the artefact’s contribution to a given value must have been intended by the designer(s) of the artefact, who designed the artefact with said function in mind. They illustrate this idea by contrasting the dyke example with the example of a knife (van de Poel and Kroes 2014):

Dykes are thus *designed for safety*. The function of a knife is cutting; cutting of, for example, bread may be instrumental to a final value like health or survival or human-well-being. However, the attainment of such final values neither is part of the function of knives nor have normal knifes been designed to achieve such final values.

The contrast of a knife with the example of a dyke is supposed to bring out the importance of design intentions for an artefact’s embedded value: (a) dykes contribute to a value (safety) and (b) they are designed for that; hence, they embody the value safety. In contrast, though normal knives contribute to a value (health), thus satisfying (a), but they are not designed for health (thus they do not satisfy (b)), and so they do not embody that value. The importance of design intentions in IHAVE will play a central role in my criticism of the account in the sections to come.

IHAVE thus presents us with the following picture. Values are grounded in the physical characteristics of the object, which determine, as in the case of the dyke, whether the artefact contributes to the value, as well as extrinsic properties of the artefact, namely what might be called its intentional history. An artefact’s intentional history is comprised of the things the designer intended the designed thing to do (e.g. to protect people from floods, in the case of a dyke). Hence, the artefact embodies a given value in virtue of being designed to contribute to that value—the artefact’s intentional history is a necessary component in determining the artefact’s embedded value.

This sketch of IHAVE leaves open the sense in which an artefact is thought to contribute to a value. Van de Poel and Kroes do not describe the contribution relation in a formal sense, though their examples provide a reasonably good intuitive picture (think of the dyke, again). In any case, it does not matter for this paper how the contribution relation is understood.

## Two Problems with the Intentional History Account of Value Embedding

IHAVE makes an artefact’s intentional history necessary for the question of which value it embeds. This fact gives rise to epistemic uncertainty about the values embedded in given artefacts, and it implies that embedded values cannot change. Thus, there are both epistemic and metaphysical worries with the intentional history account of value embedding.

### Metaphysical Problems

Consider the metaphysical problems first. To see the objection consider that, according to IHAVE, artefacts will still have value in worlds without human beings because an artefact’s value does not depend in any way on its (actual) use, but on its intended use, and intended use is fixed during the design phase of the artefact. So, while humans or other intentional creatures are required to imbue artefacts with value, they are not required to uphold the embedded value. In effect, IHAVE decouples actual use of an artefact entirely from the question of whether or not the artefact embodies a value. That seems to be going too far. While an artefact’s value cannot solely be a matter of use (as the value-neutrality thesis implies), it would seem too strong to claim that the artefact’s value is not at all a matter of use. Because an artefact’s value is, implausibly, determined at its conception, I call this the *Original Sin Objection* against IHAVE.^7^

Proponents of IHAVE might embrace the original sin objection by claiming that there is nothing wrong with an artefact’s embedded value being independent of actual use. They might point to works of art in comparison. It seems plausible, for example, to suggest that the Mona Lisa is still beautiful even if there is no one to admire the painting. There seems to be nothing in principle to block this reply. However, this reply blurs the line between value embedding in technological artefacts and other artefacts, like in artworks, and natural objects. The reply raises the question of why an account of value embedding is required for artefacts in distinction to an account of value embedding for objects more generally, including artworks and natural objects. There seems to be, however, good reason to construe value embedding differently in technological artefacts and in other types of objects. This will be so especially for proponents of the value-neutrality thesis. They insist that an artefact’s value somehow depends on how the artefact is used. If opponents of the value-neutrality thesis do not wish to deny this intuition outright, they have reason to take the original sin objection seriously.

Moreover, the original sin objection raises an issue deeper than dialectical worries. As the name suggests, an artefact gets its value at its inception or during its original design. There are two substantial problems here. First, it is a bit unclear when the value embedding takes place. Which intuitions do count and which therefore do imbue the artefact with value? If intentions contribute necessarily to the artefact’s value, then there needs to be more clarity about which intuitions count. Technically, the account assumes a three-part embodiment relation E, which can be expressed as E(F,I,V), such that a functional property F is intended (I) to contribute to value V. If in a design process there are multiple such relata, we need to know which one ends up in the object. With multiple designers, there might even be conflicts. Thus, at the very least, one would require a better understanding of whose intentions are relevant for the embedding of a value.

Even if the previous problem could be solved, there is a deeper problem still. IHAVE cannot account for relevant types of value change. Given what we called the original sin (to wit, the designers’ intention to have the artefact contribute to a value), the value thereby embedded in the artefact cannot change (short of changing the artefacts intentional history, which is impossible). So, there is a prima facie problem for the intentional history account to explain how value change is possible. To make the objection more precise, more needs to be said about the details of IHAVE as well as the type of value change in question. One type of relevant value change is the artefact changing its value; short of an argument to the contrary, it should be possible for ‘good’ artefacts’ to become ‘bad’ artefacts and vice versa. However, IHAVE allows this type of value change only by annihilating an artefact’s functional properties. Once an artefact cannot contribute to a value V anymore, it ceases to embody V. So, the only type of value change allowed for by IHAVE is the neutralization of value. There are other types of value change, however, that are relevant but which IHAVE cannot explain.

The most relevant form of value change is a change in the artefact’s embedded value. The appropriation of technology for new purposes suggests that proper value change, say, from an artefact contributing to safety to contributing to happiness, is possible.^8^ Scholars studying the appropriation of technological artefacts for novel uses distinguish between three forms of appropriation: reinterpretation (a change in semantics, with little or no physical alteration, e.g. graffiti), adaptation, defined by a change in semantic and physical use (e.g. portable cassette players used to play religious sermons) that requires a violation of intended use, and finally reinvention, where semantics, use and physical structure are changed (e.g. low-rider cars). All three forms of technological appropriation suggest that an artefact’s embedded value is malleable, but since intentional history is not affected, the intentional history account cannot explain how embedded values change with change in artefacts, use and meaning.

Another form of value change concerns changes in values themselves. Suppose that values can change in the sense that evaluative properties E that now supervene on a set of natural properties N will at another point supervene on a disjunct set of natural properties N*. Roughly, what is good today might not be good tomorrow.^9^ If this is possible, then IHAVE faces another difficulty of dealing with value change. When an artefact’s goodness is determined by the designer’s intentions then that can be translated as follows: the designer considers evaluative properties E to supervene on natural properties N and designs the artefact so that it fosters N. Suppose now that E supervenes on N* instead of N (and N* and N are disjunct). Then N is not E anymore but E*. However, the designer never intended anything about whatever value E* now supervenes on N. Given the centrality of intentions for IHAVE, the account cannot explain how embedded values change with change in values. Therefore, there are severe metaphysical concerns with the intentional history account.

### Epistemic Problems

In addition to the metaphysical problems just discussed, IHAVE faces epistemic problems. Since a given artefact’s designer’s intentions in part determine that artefact’s value, one needs to know these intentions to know the value embedded in the artefact. However, knowing an artefact’s intentional history is at the very least difficult, and, in some cases, impossible. Therefore, if IHAVE is correct, it will be difficult if not impossible to know whether a given artefact embodies some value (or any value at all). Given that the account aspires to guide engineering practice, these epistemic limitations are a severe cost. To show that an artefact’s intentional history is complicated if not impossible to determine, consider whether design intentions can be considered directly and indirectly.

Design intentions cannot be directly perceived. Inferences might be possible (which will be covered in the next section), but an agent’s intention itself cannot be perceived. Moreover, even if it were possible to perceive intentions directly, there will almost always be a temporal difference between the assessment of a given artefact’s value and the moment where the relevant design intentions were had. So, the direct perception of design intentions will likely be impossible.^10^

Design intentions are difficult if not impossible to perceive indirectly. Some argue that intentions can be inferred from observed behaviour. There are two important classes of observations that may aid indirect uncover of design intentions. First, observable features of the artefact might allow inferences about intentions. However, this class of observations makes reliable inferences about design intentions unlikely. Trying to make inferences from observable, functional features of an artefact to its intentional history is fraught with error because design choices are overdetermined. A multitude of different intentions can plausibly motivate a given design choice (e.g. the use of wood for a given component of the artefact). A choice of wood as a material for a given component of the artefact, for example, can be motivated by an intention to save material costs, to source locally, to make the object smooth to touch, to avoid health-related hazards of other materials, to appeal to taste and so forth. Without a way to narrow down the path from possibly underlying intentions to a given design choice, an indirect inference to design choices will not succeed, and the artefact’s embedded value will remain epistemically opaque. Of course, such inferences might be possible in some remote cases. However, the proponent of IHAVE should argue that it is possible more often than not.

The second class of cases where design intentions may be indirectly observable is in cases where the designer communicated or even documented their design intentions to others, for example, through design briefs or in design requirements.^11^ Design briefs, like documents or events, and documented design requirements, are used by designers to convey their intentions about the product’s functionality. Mainly because designers often aim to make their intentions clear in these contexts, it is plausible that somewhat reliable inferences about designers’ intentions can be drawn from them. Of course, access to such documents or event is not always possible, and documentation is not always preserved. Insofar as access to such documents is possible, it would seem that design intentions can sufficiently and reliably be uncovered (with a practical, rather than conceptual limiting factor). If that were the case, there might be hope to overcome some of the practical problems I mentioned and, consequently, my epistemic objection against the intentional history account of value embedding would lose some force. But the problem is not purely practical, as the following case illustrates. Suppose that a designer documents her intentions for some prototype P. A reproducer then reproduces the prototype so that P’s functional properties are kept constant. At the same time, the reproducer intends very different things for his replicas of P and also documents these intentions. As a result, there will be physically indistinguishable instances or replicas of P with very different intentional histories. Such situations abound closely enough in the real world. Whenever artefacts are reproduced by anyone that was not the inventor or original designer of the artefact, there will be a prototype with one intentional history, and replicas with different intentional histories, though the replicas’ physical features clearly do not necessarily differ (indeed, it is often the aim to keep these features as close to the original as possible). Existing differences between the intentions of P’s designer and its reproducer are, as IHAVE must hold, potentially evaluatively significant. But this commitment creates the epistemic problem of determining which intuitions count for making judgments about embedded value.^12^ The epistemic uncertainty about determining whose intuitions count in assessing an artefact poses a significant obstacle for IHAVE.^13^

These epistemic objections are not intended to establish conclusively that IHAVE fails. Perhaps there are ways for its proponents to argue that combinations of design choices enable more reliable inferences to design intentions, hence battling the epistemic imperceptibility objection. However, such work has not been done yet.^14^ What is more, there seems no plausible reply to the metaphysical objections raised above. Therefore, even though this is no rebuttal of the intentional history account yet, the preceding discussion of the problems of the account provides strong reason to investigate an alternative.

## The Affordance Account of Value Embedding

It is time to introduce what I call the affordance account of value embedding as an alternative to IHAVE. The account finds empirical support in ecological psychology and, recently, anthropology and will be strengthened with philosophical distinctions. Having sketched the account and shown its empirical foundations, I will argue that it is superior to the intentional history account of value embedding.

In order to provide a full sketch of the account, let us briefly go back to the core question of the article. The core question about value embedding is: when (if at all) does an artefact possess value? The answer to this question bears practical implications. If artefacts embed values, then responsible engineers need to be careful not to embed the wrong values in their products. Several approaches aiming at responsible, ethical product development have been discussed in the literature (van de Poel 2016; Stilgoe et al. 2013). If artefacts embed values, then these approaches presuppose that there is a way for engineers to determine what values their product will embody reliably.

Here is a first gloss of the affordance account of value embedding: an artefact embodies value if and only if it enables valuable actions; the enabling of such action is what we may have reason to promote. I will now develop this gloss into a theory. To do so, I will rely on two crucial concepts: *affordances* and *response-dependent properties*. Affordances are relational properties of objects that make particular action likely, given some circumstance. Response-dependent properties are properties of objects that depend on the objects’ intrinsic and extrinsic attributes. I will show that an object’s affordances are response-dependent properties. This allows us to say that objects embed value because of their intrinsic properties while also taking into account how an artefact’s interactive effects, which depend on its extrinsic properties, contribute to its value (which the IHAVE account could not do). I will have to say more about the nature of affordances and response-dependent properties below. For now, note that artefacts can have instrumental value: they afford some action that may in itself be valuable, and the artefact is valuable in affording to bring it about. Artefacts also embody final value. Just like actions like caring for someone’s health and helping someone’s happiness can be extrinsic final values, affording safe behaviour or affording creativity can be extrinsic final values. In each case, we have reason to value (to wit, bring about or hinder) such affordances as extrinsic, final values.^15^

More precisely, the affordance account of value embedding goes as follows:

Affordance Account of Value Embedding: Artefact x embodies value V iff x affords to a set of subjects S in conditions C an ability A and there is reason to positively respond to A (positive value), or there is reason to negatively respond to A (negative value).

The affordance relation will be interpreted as a response-dependent property below, and I will show that it is both an objective property of the artefact and a bearer of value. Therefore, it will follow that artefacts can embody values. To illustrate, an assault rifle like the AK47 affords subjects the ability to kill easily. This means that the AK47 is such that there is a significant probability that a subject succeeds in killing if the subject chooses to do so. Enabling killing, however, is a negative final value and we have moral reasons to oppose it. Therefore, the AK47 is morally bad.

We should now be able to see that the affordance account of value embedding fares very well against the desiderata derived from the discussion of IHAVE. To begin with, the account shows how artefacts can embody values that were not intended by the designer since affordances are (metaphysically) independent of intentions. Hence, one can make sense of various forms of appropriation and the account avoids the counterintuitive case of two physically identical objects differing in value. Second, the account makes it plausible that embedded value can be reliably detected. To determine what value an artefact embodies, one needs to determine what actions it affords. Though I am not claiming that this is always easy (compare: it is not always easy to determine what one’s abilities are), there are no principled reasons against succeeding as in the case of the intentional history account of value embedding. Third, the account makes it possible for embedded values to change. A full explanation of this feature will have to wait for another paper, but the general idea is that when either circumstances or abilities change, the artefact affords different things and thus embedded instrumental values may change, too. Moreover, when values themselves change, then what the artefact affords may cease to be finally valuable, and other affordances might become finally valuable instead. Fourth, the account explains embedded values in relation to human behaviour. The latter condition is intended to capture the intuition, presumably shared by proponents of IHAVE, that the embedding relation in the case of artefacts does relate to human use.

Given that the affordance account of value embedding satisfies several plausible desiderata, it appears to be a superior alternative to the intentional history account.

At this point, it is worthwhile to point out two routes for defending the affordance account of value embedding. The ambitious route would be to show that artefacts have value and then to show that all values are response-dependent properties. Given the ongoing axiological discussion about the nature of values, this is not the route I wish to take. Nonetheless, it should be mentioned that it leaves open, at least in principle, the claim that the values embedded in technical artefacts are response-dependent properties, and so, even the intentional history account of value embedding might ultimately be compatible with a basic claim defended by myself.

In any case, there is a shorter and no less attractive route to defending the affordance account of value embedding based on the following argument:

1. Artefacts embody affordances.
2. Affordances are response-dependent properties.
3. So, artefacts embody response-dependent properties. (from 1, 2)
4. Response-dependent properties are values.
5. Therefore, artefacts embody values. (from 3, 4)

The argument is deductively valid. I will now defend its soundness.

### Artefacts Embody Affordances

Artefacts embody affordances. Though I believe that this claim requires little defence, it requires clarification. Use of the concept of an affordance originates in Gibson’s ecological psychology (Gibson 1977)^16^:

The affordances of the environment are what it offers the animal, what it provides or furnishes, either for good or ill. The verb to afford is found in the dictionary, but the noun affordance is not. I have made it up. I mean by it something that refers to both the environment and the animal in a way that no existing term does. It implies the complementarity of the animal and the environment.

According to Gibson, objects as different as surfaces and substances have affordances such as fall-off-able (a cliff), eat-able (an apple), as well as events, such as cook-with-able (an event such as a fire). Gibson also already mentioned that artefacts, such as a seat, have affordances (Gibson 1977):

We call it a seat in general, or a stool, bench, chair, and so on, in particular. It may be natural like a ledge or artificial like a couch. It may have various shapes, as long as its functional layout is that of a seat. The color and texture of the surface are irrelevant. Knee-high for a child is not the same as knee-high for an adult, so the affordance is relative to the size of the individual. But if a surface is horizontal, flat, extended, rigid, and knee-high relative to a perceiver, it can in fact be sat upon.

Evidently, the affordance an object embodies depends partly on its physical structure (e.g. its horizontal, stable surface) but also on properties of the subject that uses the object (e.g. the subject’s height). Don Norman, who introduced the concept of an affordance to the design community, further emphasized the relational nature of an affordance (2013):

A chair affords (‘is for’) support and, therefore, affords sitting. Most chairs can also be carried by a single person (they afford lifting), but some can only be lifted by a strong person or by a team of people. If young or relatively weak people cannot lift a chair, then for these people, the chair does not have that affordance, it does not afford lifting.

As these quotes illustrate, an artefact’s affordances depend on the artefact’s physical properties in combination with contextual factors (including the subject’s properties). In a straightforward sense, then, whatever an artefact, or any object in general, allows you to do with it, is its affordance. The chair, in Mead’s words, ‘invites us to sit down’ (Mead 1962). Of course, the chair does not act, but it affords instead.

Recent moral anthropology provides support for the usefulness of thinking about artefacts as embodying affordances. Anthropologists have recently taken what they claim to be an ‘ethical turn’ and begun to systematically investigate the nature of morality (cf. Klenk 2019 for an overview). A common finding is that people interpret a wide variety of seemingly unconnected phenomena in ethical ways. Keane (2016) argues that these phenomena are best seen as ‘ethical affordances’ because they afford an ethical experience to subjects. For example, a torn-apart cloth given as a gift among the Sumba is interpreted as a serious insult (Keane 2016). Physical properties of the artefact (being torn apart) partly constitute what it affords: namely, in this case, interpretation as an insult (and the corresponding ethical judgement).^17^ Thus, the claim that artefacts embody affordances can be shown to be apt and useful in empirical investigations of morality. This should lend further support to premise 1.^18^

However, according to some, technical artefacts are not merely physical objects, but objects with a function (Kroes and Franssen 2015). Accordingly, an artefact is constituted by its physical and functional properties. Since artefacts are therefore constituted by objective, physical properties as well as subjective, functional properties, they have a ‘dual nature’ (Kroes 2012). For example, a screwdriver is a screwdriver partly in virtue of its objective physical properties (e.g. made partly from steel) but also in virtue of people using it as a screwdriver. The worry might be that non-artefacts bear affordance, but that artefacts do not.^19^

This objection can be dispelled. Artefacts bear affordances either way. If whatever properties the artefact has in common with non-artefacts bear the affordance, then artefacts bear affordances. If whatever sets the artefact apart from non-artefacts that bears the affordance, then artefacts bear affordances, too.^20^ So, in either case, premise 1 is vindicated.

### Affordances Are Response-Dependent Properties

Affordances should be construed as response-dependent properties. I define a response-dependent property as follows:

Response-Dependent Property: Property p of object x is a response-dependent property iff p depends on attributes of x and contextual attributes C.

Apart from defending this claim, I will show that response-dependent properties are objective properties. Their existence does not depend on being taken up by subjects, and therefore, an artefact’s value does not depend on use (therefore rebutting the value-neutrality thesis).

A prominent question in the literature on affordances is whether they depend on being perceived, attended to, sought out, used or otherwise engaged with by a subject to exist or not. A sub-question is what the precise conditions are that determine the nature of an affordance. The discussion sometimes focuses on exegetical analysis, debating, for example, whether Gibson’s concept of affordance was coherent. I am not interested in exegetical debate. Instead, I wish to argue that we should conceive of affordances as response-dependent properties by showing that the view is coherent and that it helps us make sense of value embedding.

To begin with, one might be tempted to construe affordances as secondary qualities, rather than response-dependent properties. For example, Gibson (1977) indicated that ‘to be graspable, an object must have opposite surfaces separated by a distance less than the span of the hand.’ This seems to suggest that affordances are secondary qualities: the object’s being ‘graspable-with’ depends on it being graspable by a subject. The point is significant. If affordances are secondary qualities, then the value-neutrality thesis would plausibly be true since the value of an artefact depends on it being perceived or used in a certain way.

We need not concede, however, that affordances depend on actual responses and so we can rebut this possible objection by proponents of the value-neutrality thesis. Mead’s chair, for example, invites us to sit down; it affords sitting, whether or not we sit down. Hence, it makes more sense to say that an object embodies an affordance in virtue of a potential behaviour response.

More precisely, given background circumstances C, if an organism O can at time t engage in the event that qualifies as a doing or a happening M and M involves artefact x, then x is, at t, an affordance bearer with manifestation M relative to O in the circumstances C (cf. Scarantino 2003: 958). Understanding affordances as response-dependent properties captures the essential complementarity between artefacts and the environment of their use. For example, a post-box is letter-mailing-with-able only given some background knowledge that allows subjects to distinguish the post-box from a similarly looking trash can (cf. Knappett 2004). Thus, artefact x has affordance A in virtue of its physical properties relative to a user’s potential behavioural response or ability. Of course, there are several open, and intriguing, questions about the nature of abilities, on the one hand, and their relation to affordances, on the other (cf. Scarantino 2003). To introduce the affordance account of value embedding, however, only the latter question is of immediate interest. An affordance is not defined by making specific outcomes more likely, but by making specific outcomes more likely given the circumstances provided that the subject aims to bring about these outcomes.^21^

The fact that affordances are relative to contextual factors is not a threat to understanding affordances as objective properties. For one, whether or not a given artefact embodies an affordance is epistemically objective. That is, in principle, it does not depend on the observer’s perspective to ascertain whether or not the artefact bears the respective affordance. Moreover, there is no reason to deny that affordances as construed here are metaphysically objective (pace Kroes and Franssen 2015); although their triggering conditions mention subjective elements (e.g. the intention to use the artefact), their existence does not.

Prominent philosophical accounts of response-dependent properties support the claim that response-dependent properties are objective. Pettit offers such an account when he writes that ‘the objects posited exist and have their character fixed independently of the dispositions of participants in the discourse to assert and believe things about them’ (Pettit 1991).^22^ The crucial distinction is as follows. When something red is defined by looking red to normal observers in normal circumstances, then that means that in normal circumstances, normal observers will experience the object as red. It does not entail, however, that the thing looking red is what makes the thing red (Brynjarsdóttir 2008; Jackson and Pettit 2002). So, a thing may be red even if no one perceived the object as being red.

Similarly, an artefact’s affordance might never be taken up. Consider an AK47 printed by a 3D printer left on some uninhabited, remote planet: though it is shoot-with-able, no one might ever shoot with it. It still has the affordance, because, if one were to aim to do so, in the right circumstances, one can shoot with it. Examples closer to home are not hard to find. Cultural knowledge is often required to make use of an affordance. Henrich (2016), for example, discusses the importance of cultural learning for tool use (or even tool-recognition). Europeans stranded in today’s Greenland were unable to use local tools for hunting and fishing because, among other things, they lacked knowledge of the proper techniques. Some tools, for instance, were hunt-with-able; it is just that the Europeans could not take up the affordance. The sense in which affordances exist without being triggered is the same sense in which paranoia exists for someone that never was in a confined space: a disposition to panic in a confined space exists independently of being triggered. Therefore, affordances are response-dependent properties, and as such objective properties of the artefact.

### Response-Dependent Properties Are Values

Having argued that artefacts are affordances and that affordances are response-dependent properties, I will now show that affordances thusly construed can embody values. Affordances can be both extrinsically instrumentally and extrinsically finally valuable. Therefore, affordance bearers, including technological artefacts, can be extrinsically finally valuable.

As we have seen, the affordance account does not construe values as response-dependent properties per se but instead says that the response-dependent properties of artefacts are valuable. This is a feature rather than a bug: it leaves an open question in value theory about what values are and is thus, in principle, compatible with any account of value.

I propose to view affordances as similar to actions such as helping or encouraging and to ask whether the affordance is instrumentally or finally valuable, or both. Moreover, we should think of the value of an artefact’s affordances in comparison with the value of dispositions. Dispositions can be both instrumentally and finally valuable. For example, a disposition to seek novelty is instrumentally valuable relative to the final value of happiness (Oerlemans and Bakker 2014); at the same time, a disposition for novelty-seeking may be finally valuable. Likewise, a disposition to be truthful can be instrumentally valuable for several things, but it seems finally valuable as well. Note that the value of the disposition depends on the disposition being triggered only in the case of instrumental value, not in the case of final value. For a disposition to novelty-seeking to be instrumentally valuable, one needs the disposition to be triggered. Finally, valuable dispositions, however, need not be triggered to be valuable.

This is the sense in which an artefact’s affordance results in the artefact embodying that value. An artefact may be part of the enabling conditions (similar to a disposition) for certain actions or events, given a set of contextual factors (including the subject’s properties).^23^ This enabling can be of instrumental and final value. A lazy chair’s inviting cushions make it instrumentally valuable to happiness. At the same time, the enabling of comfort may be a final value. Going back to the earlier example of an AK47, it can be seen that the rifle is of negative value because it enables killing in a broad range of circumstances. Thus, to identify the value of an artefact, we have to ask what actions or events it affords and what their value is. Importantly, it is not the action or the event that embodies the value we are interested in (though it might, too), but the affording of said action or event (compare: it is not (only) the action of helping we can evaluate but also the disposition to act in such a way). Since artefacts embody affordances, artefacts embody values. Therefore, the argument for the affordance account of value embedding is sound.

## Answering Objections against the Affordance Account of Value Embedding

Before I conclude, I aim to strengthen and clarify the account by addressing how two important objections to the account can be overcome.

A first possible objection to the affordance account of value embedding is that it implies that values are embedded in affordances, or the actions they afford, but not in the artefact itself.^24^ If that were true, the account would fail to show how artefacts embody value. However, since what is valuable is the affording of an action, not the action itself, it can be maintained that the affordance and its value is an objective property of the object. A variant of this objection is that the necessity of contextual factors (e.g. a subject’s background knowledge) for the question of whether the artefact embodies values suggests that an artefact’s value is ultimately grounded in just that: contextual factors, not the artefact itself. Two replies defuse this objection. First, there is no logical priority between the physical properties of an artefact and contextual factors outside of it when it comes to the question of value embedding. Both are necessary conditions for an artefact to embody a value. Second, if there were a way to establish such a priority (to wit, to determine on which properties the value supervenes), then a similar objection could be raised about the intentional history account of value embedding. In the case of the intentional history account of value embedding, intentions not only partly determine an artefact’s value but also the fact that the object is an artefact. It would be odd to say, however, that the artefact’s value really depends on intentions, not on the artefact itself. Thus, by consistency reasoning, I believe that the objection fails.

A second objection can initially be answered, but points to further questions that need to be worked out. To begin with, one might object that the account breaks down when considering fringe cases. Suppose, for example, an AK47 was shot into outer space, never to be used by humans again.^25^ Would the affordance account of value embedding imply that, because it will not allow for any abilities to bear fruits, the artefact loses its value? Not necessarily. The artefact certainly does not lose its affordance because an artefact’s affordance is independent of being engaged with by humans, as discussed above. Whether the object loses its value, however, depends on providing more details about value embedding according to the affordance account. Above I suggested that a given artefact possesses a vast number of affordances, which suggests that the artefact embodies a vast number of values. So, initially, I would say that the fact that the AK47 in space still affords to kill (after all, ascribing an affordance is to use a conditional predicate), it has whatever value we bestow on the enabling of killing (or, recalling the connection to the capability approach noted above, whatever value we bestow on enabling the capability of killing). In this perhaps rather superficial sense, the objection can be answered.

However, the problem cuts deeper. To illustrate, consider the movie *The Horribly Slow Murderer with the Extremely Inefficient Weapon* (Gale 2008). It depicts a murder with a spoon, which, unsurprisingly, takes many years. The right thing to say is that spoons do afford to kill. However, the triggering conditions to manifest this affordance of a spoon is exceedingly, comically unlikely (which is why the movie is absurd). Clearly, the mere fact that an AK47 on earth, an AK47 in empty space, and a spoon afford killing should not imply that they are equally valuable: they do not embody the same value to the same degree. Suggesting otherwise would lead to an oversupply of values, and it would be a point against the affordance account of value.

We can see at least the beginning of a solution to this problem by distinguishing or grading an artefact’s value relative to tacitly understood normal circumstances or contextual factors (including subjects’ properties). That is, what is relevant for an artefact’s value is whether, in normal contexts, used by normal subjects, the artefact affords certain actions or events. There are two steps to make this a bit more precise. First, one can take the set of affordances of a given artefact x and try to identify a (proper) subset of relevant affordances for evaluation. Second, one realizes that affordances must be highly specific and that affordances such as, roughly, ‘easily-killing-with-able-in-all-weathers’ are of a different value than affordances such as ‘hard-killing-with-able-only-on-sunny-days.’ Hence, since empty outer space is, plausibly, not a relevant circumstance to judge an artefact’s value, the AK47 in outer space does not lose its affordance, but plausibly its value. Relatedly, since a spoon affords to kill only in highly contrived circumstances, in comparison with, for example, an AK47, the spoon has less negative value, if any at all. So, by accompanying judgments about an artefact’s affordances with judgments about the triggering conditions of the affordance, we should be able to arrive at reliable value judgments about embodied value.

More generally, to establish value judgments that depend on an artefact’s affordance, it seems pertinent to inquire into three main questions: first, how many people will have the ability to do p, given artefact x? Second, how likely will it be that, in normal circumstances, these people will succeed at doing p, if they are aiming to do p? Finally, how normal are the circumstances in which this happens? These questions are separate from the primary question of whether we have reason to promote or avoid the enabling of p.

Of course, this puts much pressure on the notion of relevancy and the specificity of affordances in terms of normality. Unfortunately, philosophical history shows that there is little hope of establishing a reductive, non-normative account of either notion. That need not make us despair. Both notions are if defined at all, defined tacitly relative to practical interests. This is the context in which the affordance account of value embedding is primarily required. One might, therefore, be hopeful that a tacit definition will suffice.

## Conclusion

The paper defended a novel account of how artefacts can embody value, the affordance account of value embedding, based on novel objections against the prominent intentional history account of value embedding (IHAVE). An upshot of the paper is that the value-neutrality thesis is false. Further work should aim to sharpen the notion of normalcy and relevance required to prove further details on the affordance account of value embedding and to demonstrate how the affordance account of value embeeding can account for value change. Finally, it should be mentioned that the affordance account of value embedding is ultimately not incompatible with IHAVE. Even if design intentions imbue value, affordances might, too. Nonetheless, given the problems identified for IHAVE, I suggest that future efforts should be directed at developing the affordance account of value embedding.

Importantly, the affordance account of value embedding promises valuable practical upshots for engineering design and the philosophy of technology. As design for value has become almost a paradigm in the philosophy of technology, especially in the European context, we need to be able to describe and evaluate an artefact’s value accurately.^26^ The affordance account of value embedding shows that value is at stake in the design of a product, not just in its use. Therefore, it supports the urgency of design for value approaches. Moreover, the affordance account provides a link to the design community through the concept of affordance, thus encouraging interdisciplinary understanding, and it implies a naturalistic methodology for measuring embedded value, thus enabling a sound methodology for the design for value approach. For engineers aiming to design for value, the affordance account already indicates a simple recipe: measure which actions a given artefact makes likely given a context (for which we can build on social scientific tools) and then evaluate whether these affordances are legitimately desirable (for which we have normative ethics). Therefore, apart from giving urgency to the design for value paradigm, the affordance account of value embedding will help to put it on a secure footing, both theoretically and practically.

## Footnotes

1 I will use the expressions an artefact ‘embodies value’ and ‘a value is embedded in the artefact’ as synonyms. Also, I assume that ‘x embodies y’ just means that ‘y is part of x’ and that it is equivalent to say that ‘Hans is kind’ and that ‘Hans embodies kindness.’ The question about value embedding therefore becomes what it takes for artefacts to be valuable, i.e. good, bad, safe and so on.

2 Verbeek is one of the most prominent proponents of the view that artefacts have something like moral agency. Though some might interpret this as an ascription of agency to artefacts, Verbeek (2014) seems to suggest that we should rather revise the concept of agency to so that it can correctly be applied to humans and artefacts.

3 For a comparison of my account of value embedding with accounts that locate artefact-value in some (revised) notion of moral agency, based on a relational ontology (Verbeek’s and also Ihde’s work (e.g. Ihde and Malafouris 2019) are good examples), see Peterson and Spahn (2011). I omit this perspective in this paper because it would require a more extensive discussion of, among other things, conceptual engineering that I cannot hope to address adequately in this limited space.

4 Sometimes instead of using the acronym IHAVE I simply write of ‘the intentional history account.’

5 It is an interesting question how to interpret the modality of IHAVE’s causal-contribution condition, though I will not pursue it in this paper.

6 In contrast of an extrinsic property is an intrinsic property. An intrinsic property of an object is held by the object necessarily. In the language of possible worlds, the object has this property in all possible worlds. Van de Poel and Kroes’s contention that only extrinsic final value can be had by artefacts depends on the assumption that we can have the same artefact with differing intentional histories. If that were impossible, then an artefact with a different intentional history would be a different artefact and it could be consistently claimed that it has intrinsic final value. This point can be illustrated in relation to Moore’s theory that a kind of value is intrinsic if and only if, when anything possesses it, that same thing or anything exactly like it would necessarily or must always, under all circumstances, possess it in exactly the same degree (Moore 1922: 265). But when the intentional history of the object counts towards its constitution, then objects with differing intentional histories will just be different objects.

7 On first mention, I capitalize and italicize new terms of art, such as the original sin objection, but do not emphasize in subsequent mentions to increase readability.

8 Thanks to Matthew Dennis for suggesting cases of appropriation.

9 In a metaphysical sense, not just in an epistemic sense of ‘deeming’ something to be good today and bad tomorrow.

10 The Knobe effect may further corroborate the epistemic problem for IHAVE; see (Knobe 2003).

11 Thanks to Ibo van de Poel for pressing this point in defence of IHAVE.

12 Importantly, I am not making a general claim about the impossibility of uncovering intentions. My objection is limited to uncovering design intentions. To underscore this point, note that designers may fail to translate their design intentions into functional properties. In such cases of mismatched intention property sets, it is exceedingly hard to infer from properties to intentions. This is all I need for my objection.

13 Proponents of IHAVE might be tempted to reply that the focus should not be on intentions as much but on history to avoid this objection. That is, in judging an artefact, all actions and events that played a role in its creation plausibly should play a role in our moral evaluation (Thanks to Ibo van de Poel for raising this suggestion). However, this reply raises the issue of keeping apart value judgments about intentional action in a design process from judgments about intentional history. An artefacts’ history might seem important, but that might be due to the fact that judgments about an artefact’s embedded value might be inflections of value judgments related to actions that were part of the creation of the artefact.

14 Proponents of IHAVE can develop what might be called the ‘Trust-the-engineer’ and the ‘predestination’ reply. The former would require showing that a designer’s intentions to make artefact x contribute to value V (by means of properties p) reliably make this the case. The predestination reply would require showing that there is a sufficiently limited amount of design options to make some artefact contribute to some value V. I believe that neither reply ultimately succeeds, however, because of ubiquitous examples of failed designs (or fortuitous ones, conversely) and the multiple realizability of moral values. In any case, developing the required response to my objection would be beyond the scope of this paper.

15 Of course, which things ultimately count as finally valuable is an open question of value theory broadly understood. For this paper, however, it suffices to show that the affordance account of value embedding implies that artefacts are the things that can have such kind of value and insofar as relational properties and actions can be finally valuable; this desideratum is met. The account also neatly aligns with practical concerns: to develop ethically good products, engineers must ask what actions their product will afford and whether enabling these actions is morally valuable.

16 The rationale for adopting the concept of affordance for the philosophy of technology is fruitfulness: work in other disciplines has shown that the concept is explanatorily useful, and this paper argues that this is also the case when thinking about value embedding in the philosophy of technology.

17 At this point, post-phenomenological attention to the mediating role of technology should be in favour of the affordance account of value embedding; see Verbeek (2006).

18 Apart from anthropologists and designers, archaeologists (e.g. Knappett 2004), and media scholars (e.g. Schrock 2015) find affordance a useful concept to work with. My rationale for adopting the concept for the philosophy of technology is explanatory fruitfulness. Of course, one needs to be aware of potential translation errors. Hence, I am explicit in my explication of the concept, and in doing so, I go beyond the literature from which I borrow. I thank an anonymous reviewer for prompting me to clarify this. To give just one example, there are limitations to the usefulness of Keane’s account for the purposes of explaining value embedding. Keane leaves open the question of what explains when a thing gets taken up as an affordance. Therefore, the account does not offer much help for designers that want to influence what things their products afford. Moreover, Keane explicitly says that any experience can be taken up as ethical. So, is there no constraint on what can count as ethical? At this point, it should be clear that Keane constructs his account of an affordance along the lines of a Lockean secondary quality. Accordingly, for an object x to have property p is for some observer S to perceive x as having p. By extension, some artefact x embodies value V if x affords V, that is if some observer perceives x as having V. So, if any feature of an object can be taken up as ethical, there are no constraints on what value an artefact can afford. This will not do for the account of value embedding.

19 Heft (1989) argues that, based on Gibson’s notion of affordances, affordances of artefacts differ from non-artificial physical objects. My account frees affordances to depend on cultural information, too, and so it avoids Heft’s objection. Cosentino (2019) has recently argued that a distinction should be maintained between a type of affordance that relates to cultural factors and another type that relates to species-specific physical factors to avoid confusing distinct types of knowledge that arise from both types. What I say in what follows is compatible with her account.

20 See Kroes and Franssen (2015) for discussion.

21 Note that an affordance resembles what Robeyns (2005: 99) calls ‘conversion factors’ in the context of the capability approach (cf. Nussbaum 1988, 2004; Sen 2011) to explain how goods (e.g. a bicycle) enable certain functions or capabilities (e.g. mobility). A paved road does not necessarily make it more likely that someone uses a bike, but it enables the use of a bike. The relation of the affordance account of value embedding to the capability approach merits further exploration. The pertinent point for now, however, is that affordances enable certain outcomes, conditional on the subject’s aims and contextual factors.

22 According to Johnston’s (1989) account of response-dependent concepts, a response-dependent concept X is the concept of a disposition to x. Following Johnston, most philosophers have discussed response-dependent concepts Jackson and Pettit (2002); Pettit (1991) and some assumed that there is a straightforward translation scheme between response-dependent concept and properties Clercq (2001). I will not discuss this point here.

23 ‘Enabling’ should not be understood in terms of ‘making likely’. I return to the point below.

24 Thanks to Ibo van de Poel for raising this objection.

25 Thanks to Martin Peterson for raising this objection.

26 Thanks to an anonymous reviewer for prompting me to clarify this point and for suggesting the idea that design for value is almost a paradigm in contemporary European philosophy of technology.
